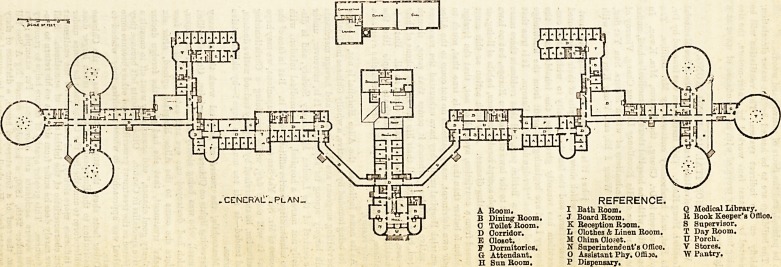# Asylum for the Insane, Waterbury, Vermont

**Published:** 1892-04-16

**Authors:** 


### HOSPITAL CONSTRUCTION.

### ASYLUM FOR THE INSANE, WATERBURY, VERMONT.

One of the latest asylums for the insane in the United States is now in process of construction for the State of Vermont, in the town of Waterbury. As modern asylums go it is not a large one, and is intended chiefly for chronic patients, which accounts for the undivided circular wards and the associated dining-rooms. It is of the pavilion type, but with the pavilions more separated than usual, the connecting corridors being of some length and only one storey in height.

The buildings comprise a three-fold group, the central, or administration building, and two wings. The long frontage is to the east, the rear faces the west, and the rooms are so arranged that there is scarcely a patients' room, and few others, which does not have either the morning or afternoon sun.

The central group comprises an official building in front, with the superintendents, physicians, and stewards' offices and rooms, the chapel building behind it having store-rooms in the basement and first floor, and the chapel on the second floor; back of the chapel is the general kitchen with its accessories, while in the rear of all is the boiler house and laundry.

The two wings are substantially alike, that to the north being intended for men, and that to the south for women. Each group comprises eight wards in six buildings, connected to each other and to the centre by one-storey corridors. The wards are thus sufficiently separated to comply with the latest demands of sanitary science, and to guard greatly against the spread of fire, and yet are so connected as to facilitate communication in a cold climate and secure economy of construction and administration.

A description of one wing will apply also to the other.

The building nearest to the centre is three storeys high, having a ward on each floor for patients with their attendants. Each of these wards is complete with its day-rooms, two small associated dormitories, bed-rooms, dining-room, bath and toilet rooms, &c., and two staircases.

From the farthest corner of this building a one-storey corridor leads towards the west to a building for the criminal insane. This building has separate entrances, as indeed have all the others. It is two storeys high, having a complete ward arranged for 12 patients on each floor.

Another one-storey corridor also leads from the first-mentioned ward in the direction of the long axis to a group of three circular buildings, intended especially for chronic patients. Each of these buildings is two storeys high, and is arranged for 25 patients, the first floor being used for a day-room, and the second floor for an associated dormitory. Rectangular connecting portions give room for the attendants, for bath-rooms, stairs, &c. Two sun-rooms enclosed by glazed sashes are provided in connection with these circular wards. An asso ciated dining-room, large enough for the whole 75 patients of the chronic wards of one wing, is placed adjacent to the corridor. This also is but one storey high.

The total accommodation of each wing is 189, or 378 patients for the whole building. Probably by a little pressure 400 could be taken care of.

The walls of the building are of local brick of good quality and colour, resting on an under-pinning of granite, laid as broken ashlar. All other dressed stone is also of granite, while the foundations are of local stone. The exterior brick walls are sixteen inches thick, and the interior walls, also of brick, are for the most part eight inches thick. The outside walls are hollow to maintain warmth and prevent dampness, and all plastering is done directly upon the brick. The floors are deafened, and the spread of fire retarded by a thick layer of plaster between the under and upper boards. The roofs are slated. The inside finish is very simply designed of pine, to be painted, and the floors of native hard wood.

The buildings are to be heated by hot-water circulation, chiefly on the indirect system. The ventilation will be assisted by exhaust fans worked by water-motors, the site being fortunately provided with an abundance of water at a high pressure.

## Figures and Tables

**Figure f1:**